# Use of telemedicine to combat the COVID-19 pandemic in Brazil

**DOI:** 10.6061/clinics/2020/e2217

**Published:** 2020-07-27

**Authors:** Carlos Roberto Ribeiro Carvalho, Paula Gobi Scudeller, Guilherme Rabello, Marco Antonio Gutierrez, Fabio Biscegli Jatene

**Affiliations:** I Pneumologia, Instituto do Coracao (InCor), Faculdade de Medicina FMUSP, Universidade de Sao Paulo, Sao Paulo, SP, BR; II Inovacao, Instituto do Coracao (InCor), Faculdade de Medicina FMUSP, Universidade de Sao Paulo, Sao Paulo, SP, BR; III Divisao de Informatica, Instituto do Coracao (InCor), Faculdade de Medicina FMUSP, Universidade de Sao Paulo, Sao Paulo, SP, BR

In 2019, in Brazil, respiratory diseases accounted for more than 1 million hospital admissions and cost the Brazilian’s Unified Health System (SUS) more than R$ 1 billion; in contrast, while diseases due to circulatory problems also led to practically the same number of hospital admissions, they imposed a three-fold higher economic burden. In the same year, in the State of São Paulo, respiratory diseases had a mortality rate of approximately 13% (DATASUS), which increased to 52% if the patient was on mechanical ventilation (1). Pneumonia accounted for three-fold higher hospital admissions than acute myocardial infarction, resulting in 15,137 deaths from pulmonary disease and 3,584 from cardiovascular disease, demonstrating the high impact of this respiratory disease on the health system.

The severity of respiratory diseases makes it necessary for the patient to be hospitalized, and, in more severe cases, the use of supplemental oxygen and mechanical ventilatory support. This type of treatment is preferably provided in intensive care units because it requires trained personnel and state-of-the-art materials/equipment. In epidemic situations, such as the Severe Acute Respiratory Syndrome in 2003, the flu caused by H1N1 influenza in 2009, or Ebola in 2014, the establishment of specialized centers with strategies for screening, referral, and counter references for the specialized care of severe cases proved to be fundamental. In line with this epidemiological profile, the Heart Institute of the HCFMUSP implemented, in September 2018, an Intensive Respiratory Therapy Unit with 10 beds, considered a reference for the Health Department of the State of São Paulo, for the care of patients with severe respiratory failure.

In 2020, the pandemic caused by SARS-CoV-2 led to many severe cases of acute respiratory failure, and it was realized that proper management of mechanical ventilation, by a specialized team, would be essential for the care of such patients. To expand the outreach of this model of assistance, a network of Respiratory ICUs was implemented in the State of São Paulo, interconnected by telemedicine, for the management of serious respiratory problems. Telemedicine teams working around the clock provided remote support for the network of Respiratory ICUs.

When there was an abrupt increase in the demand for ICU beds, a training platform driven by telemedicine allowed the establishment of a protective mechanical ventilation protocol, proposed by the Respiratory ICU of INCOR-HCFMUSP in partnership with the State Department of Health. After 90 days of availability, more than 125,000 users had already accessed the platform. The telemedicine-driven capacitation training was provided to the frontline professionals, doctors, nurses, and physiotherapists. Telemedicine was also instrumental in facilitating distance learning and training. Through this project, a novel virtual-reality simulator-based training was also provided to the multidisciplinary teams of hospitals in the service network. In skills-based education, where hands-on learning plays a central role, the integration of theoretical knowledge with practical application is important.

A simulation application was developed based on our current knowledge of SARS-CoV-2 and the protective mechanical ventilation protocol, proposed by the INCOR-HCFMUSP Respiratory ICU.

Telemedicine was a practice regulated by the Ministry of Health, as per ordinance no. 467 as of 03/20/2020, on an exceptional and temporary basis, to manage the COVID-19 pandemic, provided for in Art. 3 of Law No. 13,979, as of February 6, 2020. The TeleICU project, supported by the State Department of Health, was thus implemented. This project foresaw the inclusion of nine state public hospitals, representing 270 ICU beds monitored at a monthly cost of USD 7000 per hospital. Reassessment after 70 days of implementation allowed inclusion of 10 additional hospitals, increasing the number of ICU beds by 297, with the total being 567 beds supported by the project, thereby reducing the monthly cost of a hospital by 57%. The investment in this project included equipment, daily remote care of ICU cases, and capacitation and training of the staff.

Cases are discussed between the intensive care physician on the TeleICU team and the hospital's multidisciplinary team in the Respiratory ICU network in the State of São Paulo. To meet the requirements of information security and confidentiality during the discussion of clinical cases, the platform of the University Telemedicine Network or RUTE Network (2) was adopted instead of commercial Web conferencing apps, avoiding possible litigations with leakage of sensitive personal data such as results of examinations and diagnoses of patients (3).

The RUTE Network is an initiative in Brazil that aims to promote the integration of Telemedicine and Telehealth among university hospitals, medical schools, and individual clinics. This has been made possible through an information and communication technology infrastructure provided by the National Research Network (RNP), a social organization linked to the Ministry of Science, Technology, Innovations and Communications (MCTIC), which is the core of the Brazilian academic network (4). Using this technological infrastructure, telemedicine stations were developed with high-resolution dual monitors equipped with a camera set, an echo cancellation microphone, and an audio reproduction device. Videoconferencing sessions between health professionals take place on one of the monitors, while on the other, information present in the electronic medical records of the hospitals participating in the discussion is shared.

To record information such as patient history, results of laboratory tests, ventilation parameters, and conducts, a set of electronic forms was developed using the REDCap application (5). REDCap is a secure Web application for creating and managing online surveys and databases. While it can be used to virtually collect any type of data, including environments compliant with US FDA 21 CFR Part 11, FISMA, and HIPAA standards, it is specifically targeted to support online or offline data capture for research studies and operations. The REDCap Consortium, a vast network of employees, is composed of thousands of institutional partners active in more than 100 countries that use and support REDCap in various ways, with Hospital das Clinicas being one of the institutions that make up the consortium.

In addition to recording all the variables of interest, collected during the discussion of clinical cases, by safely following international standards such as HIPPA, we also opted to record all discussions as MPEG-4 files (6). This allows the possibility to revisit and review any case.

TeleICU makes this highly complex and technology-driven healthcare more accessible (7). Meta-analyses (8,9) demonstrated a reduction in ICU mortality, hospital mortality (10,11), and duration of ICU stay with TeleICU. A study in Massachusetts (12) showed a 20.7% increase in bed turnover and estimated that an efficient TeleICU program will reduce the care cost per patient in 3 months, even making up for its implementation costs.

The value addition by TeleICU, considering the initial investment, will depend on the extent to which it influences clinical outcomes, satisfaction rates for care and education, and specialization projects for professionals (9). The cost can be recovered through an increase in bed turnover, saving of human resources, and reduction of risks and errors.

As innovation becomes a constant process in any industry, such as healthcare, it has become more important than ever in this pandemic to track and manage information and ensure that resources and results are used in the most effective manner. There is no scarcity of data across the health sector, but definitely, there is a shortage of contextualization and understanding of the data being collected. In the case of this ICU network project, innovation was also sought in monitoring the indicators generated.

Medical dashboards provide a centralized visual method to contextualize data, improve visibility, and prioritize indicators, in addition to promoting transparency and helping clinicians and managers to make better decisions about the patients' care.

Implementation of the Respiratory TeleICU network has been a high-level approach to manage the pandemic caused by SARS-CoV-2. It allowed establishment of a management protocol, as well as facilitated the training and supervision of more than 19 reference teams in this service. With this, we achieved the main objective of our work, which was to improve the quality of care provided to patients using the unified health system.

## AUTHOR CONTRIBUTIONS

Carvalho CRR was responsible for the concept and protocol definition to manage ICUs remotely. Scudeller PG was responsible for the project inception and management, indicators definition and simulation model. Rabello G was responsible for the medical dashboards definition and simulation model. Gutierrez MA was responsible for the information technology infrastructure and database model definition. Jatene FB was responsible for the innovation drive.
